# Venezuelan Equine Encephalitis, Peruvian Amazon, 2020

**DOI:** 10.3201/eid3105.241694

**Published:** 2025-05

**Authors:** Marta Piche-Ovares, Maria Paquita García Mendoza, Andres Moreira-Soto, Carlo Fischer, Sebastian Brünink, Maribel Dana Figueroa-Romero, Nancy Susy Merino-Sarmiento, Adolfo Ismael Marcelo-Ñique, Edward Málaga-Trillo, Miladi Gatty-Nogueira, César Augusto Cabezas Sanchez, Jan Felix Drexler

**Affiliations:** Charité Universitätsmedizin Berlin, Berlin, Germany (M. Piche-Ovares, A. Moreira-Soto, C. Fischer, S. Brünink, J.F. Drexler); Instituto Nacional de Salud, Lima, Peru (M.P. García Mendoza, M.D. Figueroa-Romero, N.S. Merino-Sarmiento, A.I. Marcelo-Ñique, C.A. Cabezas Sanchez); Universidad Nacional de Costa Rica, Heredia, Costa Rica (A. Moreira-Soto); Universidad Peruana Cayetano Heredia, Lima (E. Málaga-Trillo); Laboratorio de Refencia Regional en Salud Publica, Loreto, Peru (M. Gatty-Nogueira); German Centre for Infection Research, associated partner Charité-Universitätsmedizin Berlin, Berlin (J.F. Drexler)

**Keywords:** Venezuelan equine encephalitis, viruses, meningitis/encephalitis, zoonoses, enzootic, genome, serology, Peru

## Abstract

We screened 1,972 febrile patients from the Peruvian Amazon in 2020–2021 for Venezuelan equine encephalitis virus (VEEV). Neutralizing antibody detection rate was 3.9%; 2 patients were PCR positive. Genome identity compared to Peru VEEV subtype ID strains was 97.6%–98.1%. Evidence for purifying selection and ancestry ≈54 years ago corroborated VEEV endemicity.

The Venezuelan equine encephalitis antigenic complex of alphaviruses encompasses 6 subtypes (I–VI), originally designated according to antigenic properties (*1*); those 6 subtypes are further divided into antigenic varieties. Venezuelan equine encephalitis virus (VEEV) consists of 4 varieties (AB, C, D, and E); subtype ID is enzootic in Central and South America (*2*). The VEEV ID transmission cycle involves mosquitoes of the genus *Culex* (subgenus *Melanoconion*); however, knowledge of vertebrate sylvatic hosts is scarce (*3*). A prior study from the Peruvian Amazon conducted in 2001–2007 using molecular and serologic techniques indicated that up to 7% of febrile cases are potentially caused by VEEV, making it one of the most relevant arthropodborne viruses regionally (*4*).

VEEV has been detected continuously in humans in the Peruvian Amazon since 1993 (*4*,*5*). In 2006, a reported outbreak of VEEV resulted in a 5-fold increase in cases (63 cases) compared with previous years (10–14 cases/year) and 2 human deaths (*6*,*7*). All samples sequenced in the 2006 outbreak belonged to subtype ID (*8*). Since 2013, no data have become available on VEEV from Peru, so it is possible that the epidemiology of VEEV might have changed. We thus investigated VEEV in the Peruvian Amazon during 2020–2021 by using molecular, serologic, and bioinformatic methods.

## The Study

**Figure 1:**
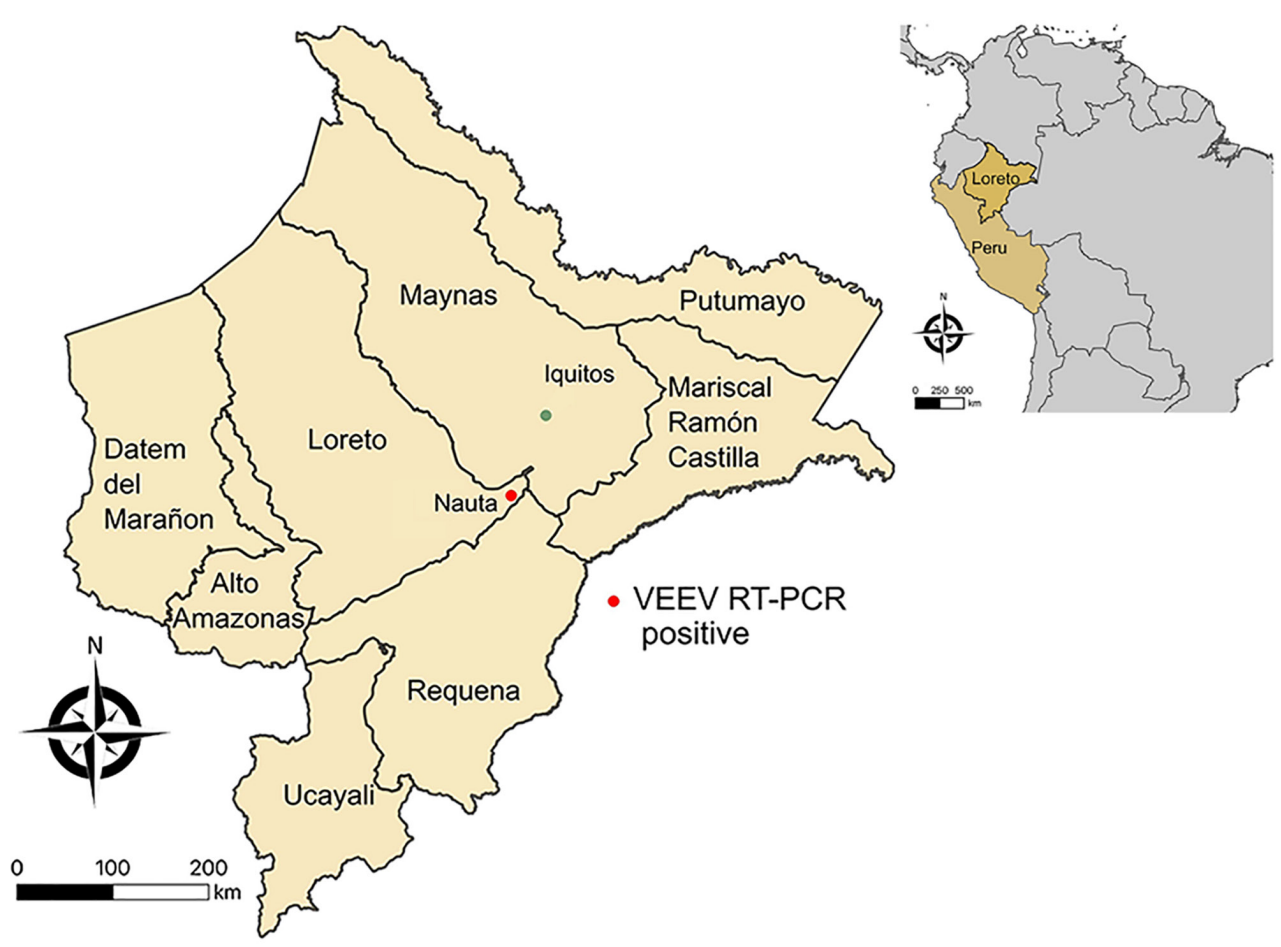
Geographic location of Venezuelan equine encephalitis virus–positive case from study of Venezuelan equine encephalitis, Peruvian Amazon, 2020. Red dot shows location of case for which metadata were available; green dot represents Iquitos, capital of the department of Loreto. Inset map shows location of Peru in South America and of Loreto department. All maps were created using QGIS 3.36.0 based on freely available maps from Bucknell University (https://hub.arcgis.com) and Instituto Nacional de Estadística e Informática, Peru (https://ide.inei.gob.pe/#capas).

We screened 1,972 serum samples from febrile humans sampled in the Peruvian Amazon as part of routine acute febrile illness surveillance in 2020–2021 during overlapping dengue virus and SARS-CoV-2 outbreaks (*9*). We tested samples for alphavirus RNA by a genuswide, nested reverse transcription PCR (RT-PCR) (*10*). Two serum samples (0.1%, 95% CI 0.001%–0.4%) tested positive for the presence of VEEV RNA. We obtained the first sample (Peru_2020) from a 74-year-old man from the district of Nauta in February 2020 (Figure 1). The patient sought treatment for headache, fever, muscle pain, and respiratory symptoms 4 days before sampling. The patient’s viral load was 8.1 × 10^5^ copies/mL, as determined by a strain-specific real-time RT-PCR (*6*), consistent with the time elapsed since the onset of symptoms. We obtained the second sample (Peru_2021) from a 12-year-old girl in January 2021. Her viral load was 1.2 × 10^3^ copies/mL, but no additional information was available. Isolation of both samples on Vero cells enabled the viral genomes to be characterized by high-throughput sequencing (Illumina, https://www.illumina.com) (Appendix). 

**Figure 2:**
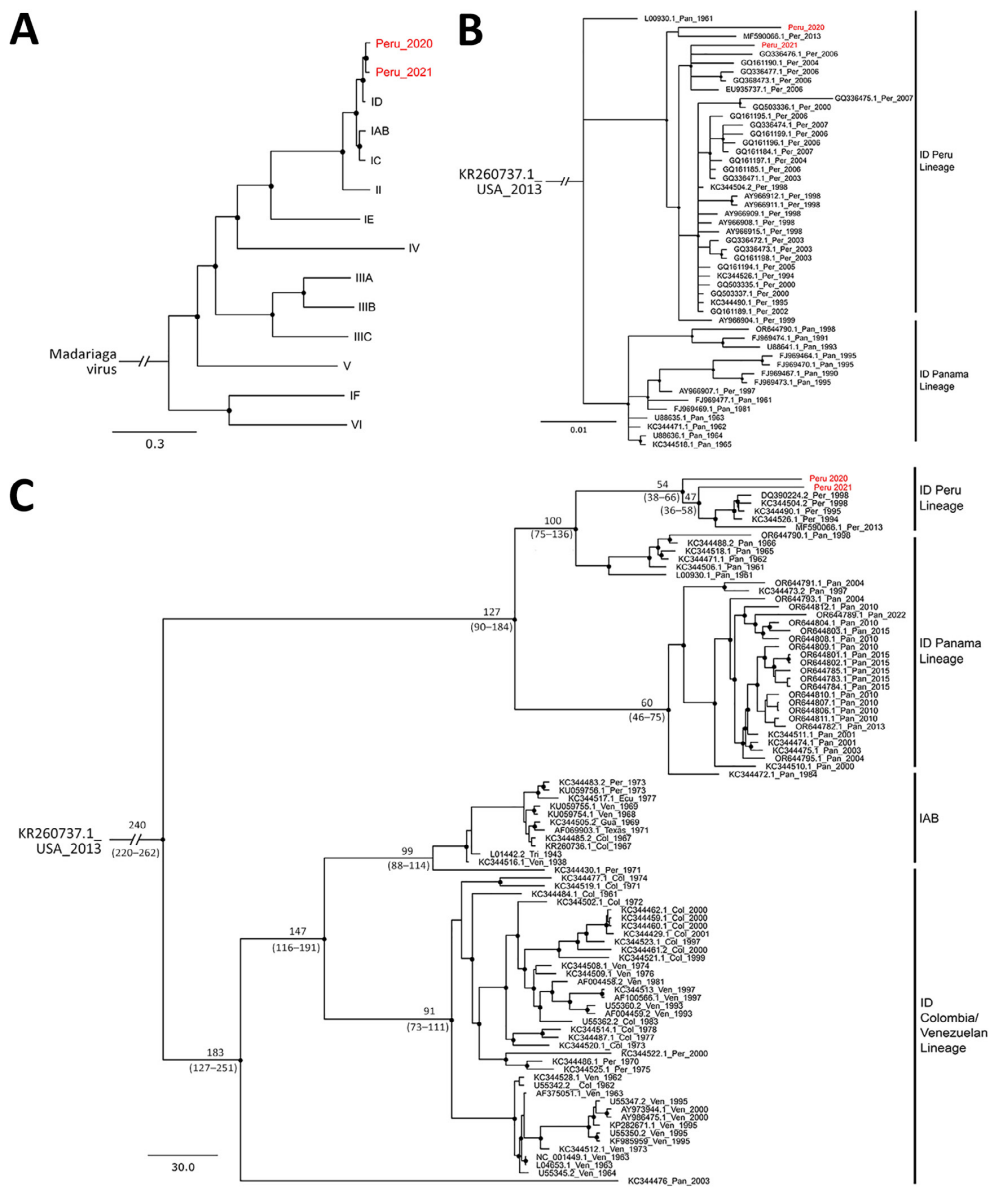
Phylogenetic relatioships of Venezuelan equine encephalitis virus (VEEV) from study of Venezuelan equine encephalitis, Peruvian Amazon, 2020. A) Relationships of VEEV from Peru (Peru 2020 and Peru 2021, depicted in red) and members of the Venezuelan equine encephalitis antigenic complex, based on the concatenated coding sequence (11,629 nt). Madariaga virus was included as an outgroup. B) Phylogenetic relationships of Peru 2020 and Peru 2021 (shown in red) and members of VEEV subtype ID Panama/Peru lineage, based on a partial sequence of the envelope glycoprotein precursor (PE2, 817 nt). Phylogenetic trees were constructed using MrBayes 3.2.6 (https://github.com/NBISweden/MrBayes/releases/tag/v3.2.6). GenBank accession number, country, and collection year are indicated for each sequence. Posterior probability ≥0.80 is indicated as a black circle in the node. C) Time to most recent common ancestor of VEEV identified in this study (Peru 2020 and Peru 2021, shown in red) and members of the ID and IAB subtypes, by number of years ago, calculated using BEAST 1.7.1 (https://beast.community), based on the concatenated coding sequence (11,629 nt). Tip dates were obtained from the sampling year following a previous phylogenetic analysis of VEEV subtype ID and IAB; no further calibration of node ages was performed to construct the tree. Times are identified at each branch by number of years ago; numbers in parentheses indicate 95% highest posterior density values in years (*11*). Col, Colombia; Ecu, Ecuador; Gua, Guatemala; Pan, Panama; Per, Peru; Ven, Venezuela.

In a Bayesian whole genome–based phylogeny, the 2 newly generated sequences (GenBank accession nos. PP700505 and PQ513527) clustered with the ID subtype (Figure 2, panel A). We also observed clustering within ID in phylogenies based on all publicly available envelope glycoprotein precursor genomic sequences of the ID Panama/Peru lineage (Figure 2, panel B). Sequence comparisons indicated that both VEEV strains from our study were closely related (sequence identity 97.6%–98.1%) to other Peruvian strains belonging to the ID lineage but not were monophyletic, highlighting the cocirculation of different strains and suggesting maintenance of that VEEV clade in the region. We estimated the time to most recent common ancestor at ≈54 years ago (95% highest posterior density 38–66 years ago) (Figure 2, panel C) and noted evidence for purifying selection that suggested a regionally stable VEEV transmission cycle (*11*) (Appendix).

To determine past VEEV infections in Peru’s Loreto department, we selected a subset of 463 samples collected during January–April 2020 and September 2020–January 2021, corresponding to rainy seasons, when vector presence is highest and transmission is most likely. Samples were also selected to be negative for dengue virus by RT-PCR and of sufficient volume to enable serologic testing. We tested serum samples first by plaque reduction neutralization test (PRNT) in a 96-well plate format at 1:40 serum dilution to neutralize ≈50% (PRNT_50_) of the plaque-forming units of a VEEV isolate from this study. We retested positive samples (53/463) by PRNT_50_ in a 12-well plate format, which enables better count of plaque-forming units, in 2-fold dilutions ranging from 1:40 to 1:320 (Appendix). The titration showed that 18 of the 463 samples (3.9%, 95% CI 2.4%–6.1%) robustly demonstrated neutralizing antibodies (Table 1). Of note, a 2006 study reported a VEEV seroprevalence of 23%, which differed from our results for unknown reasons, potentially including sampling focused on urban areas and the VEEV outbreak reported during that year (*7*).

**Table 1:** Alphavirus serologic reactivity patterns in VEEV IgG-positive serum samples from Loreto, Peru, in study of Venezuelan equine encephalitis, Peruvian Amazon, 2020*

Sample	VEEV PRNT_50_ titer	IgG indirect immunofluorescent assay result									
		CHIKV	ONNV	RRV	BFV	SINV	WEEV	EEEV	VEEV	CHIKV vlp	MAYV vlp
3216	>1:320	+	+	+	–	–	+	–	+	+	+
3270	1:40	+	+	+	+	+	+	+	+	+	+
**3393**	1:40	–	–	–	–	–	–	–	+	–	–
**3399**	1:160	–	–	–	–	–	–	–	+	–	–
**3624**	1:40	–	–	–	–	–	–	–	+	–	–
3634	1:40	+	+	+	–	–	+	–	+	+	+
**3876**	1:40	–	–	–	–	–	–	–	+	–	–
**3927**	1:80	–	–	–	–	–	–	–	+	–	–
**3940**	1:40	–	–	–	–	–	–	–	+	–	–
4140	>1:320	+	–	+	–	–	+	–	+	–	–
4294	1:160	+	+	+	–	–	–	–	+	+	+
4321	1:80	+	–	+	–	–	+	+	+	–	–
4420	1:80	–	–	–	–	–	–	–	+	–	+
**4590**	1:40	–	–	–	–	–	–	–	+	–	–
**4669**	1:80	–	–	–	–	–	–	–	+	–	–
**4747**	1:160	–	–	–	–	–	–	–	+	–	–
4768	1:40	+	–	–	–	–	–	–	+	+	+
4797	1:160	+	–	–	–	–	+	–	+	–	+

*Bold indicates monotypic reactions. BFV, Barmah Forest virus; CHIKV, chikungunya virus; EEEV, Eastern equine encephalitis virus; MAYV, Mayaro virus; ONNV, o’nyong-nyong virus; PRNT_50_, plaque reduction neutralization test with ≥50% plaque reduction considered positive; RRV, Ross River virus; SINV, Sindbis virus; VEEV, Venezuelan equine encephalitis virus; vlp, virus-like particle; WEEV, Western equine encephalitis virus.

Considering the potential for cross-reaction with other alphaviruses, we tested PRNT_50_–positive samples by using a commercially available indirect immunofluorescent assay (IFA) (EUROIMMUN, https://www.euroimmun.com). The assay was based on cells infected with VEEV along with chikungunya, o’nyong-nyong, Ross River, Western equine encephalitis, Sindbis, Barmah Forest, and Eastern equine encephalitis viruses, as well as virus-like particles for Mayaro and chikungunya viruses (Appendix). Serum samples were tested at 1:100 for IgG and at 1:10 for IgM, which is the serum dilution recommended by the manufacturer. All PRNT_50_–positive serum samples tested positive for VEEV in the IgG IFA, and we observed monotypic VEEV reactivity in 9 of 18 samples (50%, 95% CI 29.0%–71%) (Appendix Figure 1). All other samples reacted with ≥2 viruses, results expected in tropical areas where several alphaviruses cocirculate (*12*). Cross-reactivity might have affected IFA patterns, since reactivity was observed with viruses not previously reported in Latin America, such as o’nyong-nyong, Ross River, Sindbis, and Barmah Forest viruses (*13*) (Table 1). Reactivity patterns suggested a predominance of Mayaro (n = 7) over chikungunya (n = 5) virus and of Western equine encephalitis (n = 6) over Eastern equine encephalitis (n = 2) virus, highlighting the relevance of targeting Mayaro and Western equine encephalitis virus in regional diagnostic testing and targeted epidemiologic studies. 

IgG detection rates did not vary significantly by sex (p = 1.0 by Fisher exact test) but were higher in adults >30 years of age (p = 0.0001 by Fisher exact test), potentially a consequence of the 2006 VEEV outbreak (Table 2) (*7*). Adapting the IFA for IgM yielded entirely negative results, which is consistent with low levels of VEEV-specific IgM in the serum of acutely infected patients (*14*) and predominant detection of nonrecent infections in this study (Appendix Figure 2).

**Table 2:** Seropositive patients and demographic variables from study of Venezuelan equine encephalitis, Peruvian Amazon, 2020*

Category	VEEV IgG IFA positive, no. (%)	Total no. (%)
Sex†		
F	9 (3.9)	234 (50.4)
M	9 (4.0)	229 (49.5)
Age, y‡		
<15	0 (0.0)	202 (43.6)
15–29	4 (3.3)	121 (26.1)
30–44	7 (9.9)	71 (15.3)
≥45	7 (10.1)	69 (14.9)

*Statistical analyses were performed using R version 2024.04.2 (The R Project for Statistical Computing, https://www.r-project.org). VEEV, Venezuelan equine encephalitis virus. †p = 1.0 by Fisher exact test. ‡Compared in 2 age groups: 0–29 and >30 years; p = 0.0001 by Fisher exact test.

## Conclusion

Despite evidence for the medical relevance of VEEV ID in the Peruvian Amazon from a prior study (*5*) and our study, the diagnosis of VEEV is not included in the routine arbovirus surveillance panel of Peru (N°125-MINSA/2016/CDC-INS). Our study demonstrates that the lack of surveillance and diagnosis is making the infection go unnoticed.

In tropical regions, factors such as the proximity of the rainforest to urban areas, agricultural activities, and deforestation may increase the likelihood of human contact with vectors, leading to virus transmission (*14*). These factors are of particular significance in the Peruvian Amazon, where ≤96.7% of households engage in activities related to agriculture or aquaculture (https://data-peru.itp.gob.pe). This information could explain why adults are more likely to be in contact with the virus and why nonpharmaceutical interventions to combat the spread of SARS-CoV-2 do not appear to affect the transmission of VEEV (*15*).

Beyond increased diagnostics, future research should prioritize investigating transmission cycles of different VEEV subtypes to identify populations at risk and focus on potential prevention strategies, such as targeted control of vectors and identifying potential amplifying vertebrate hosts (*1*). Arboviral surveillance, including of VEEV, should be generally strengthened with a syndromic diagnostic approach, particularly during periods of increased rainfall and harvest season, when the risk of arboviral infection may be increased (*14*). 

Appendix Additional information for Venezuelan equine encephalitis, Peruvian Amazon, 2020.

## References

[1] Weaver SC, Barrett AD. Transmission cycles, host range, evolution and emergence of arboviral disease. Nat Rev Microbiol. 2004;2:789–801. 10.1038/nrmicro1006 15378043 PMC7097645

[2] Aguilar PV, Estrada-Franco JG, Navarro-Lopez R, Ferro C, Haddow AD, Weaver SC. Endemic Venezuelan equine encephalitis in the Americas: hidden under the dengue umbrella. Future Virol. 2011;6:721–40. 10.2217/fvl.11.50 21765860 PMC3134406

[3] Aguilar PV, Greene IP, Coffey LL, Medina G, Moncayo AC, Anishchenko M, et al. Endemic Venezuelan equine encephalitis in northern Peru. Emerg Infect Dis. 2004;10:880–8. 10.3201/eid1005.030634 15200823 PMC3323213

[4] Forshey BM, Guevara C, Laguna-Torres VA, Cespedes M, Vargas J, Gianella A, et al.; NMRCD Febrile Surveillance Working Group. Arboviral etiologies of acute febrile illnesses in Western South America, 2000-2007. PLoS Negl Trop Dis. 2010;4:e787. 10.1371/journal.pntd.0000787 20706628 PMC2919378

[5] Watts DM, Russell KL, Wooster MT, Sharp TW, Morrison AC, Kochel TJ, et al. Etiologies of acute undifferentiated febrile illnesses in and near Iquitos from 1993 to 1999 in the Amazon River Basin of Peru. Am J Trop Med Hyg. 2022;107:1114–28. 10.4269/ajtmh.22-0259 36162442 PMC9709010

[6] Vilcarromero S, Aguilar PV, Halsey ES, Laguna-Torres VA, Razuri H, Perez J, et al. Venezuelan equine encephalitis and 2 human deaths, Peru. Emerg Infect Dis. 2010;16:553–6. 10.3201/eid1603.090970 20202445 PMC3322018

[7] Morrison AC, Forshey BM, Notyce D, Astete H, Lopez V, Rocha C, et al. Venezuelan equine encephalitis virus in Iquitos, Peru: urban transmission of a sylvatic strain. PLoS Negl Trop Dis. 2008;2:e349. 10.1371/journal.pntd.0000349 19079600 PMC2593782

[8] Aguilar PV, Adams AP, Suárez V, Beingolea L, Vargas J, Manock S, et al. Genetic characterization of Venezuelan equine encephalitis virus from Bolivia, Ecuador and Peru: identification of a new subtype ID lineage. PLoS Negl Trop Dis. 2009;3:e514. 10.1371/journal.pntd.0000514 19753102 PMC2734058

[9] Plasencia-Dueñas R, Failoc-Rojas VE, Rodriguez-Morales AJ. Impact of the COVID-19 pandemic on the incidence of dengue fever in Peru. J Med Virol. 2022;94:393–8. 10.1002/jmv.27298 34436792 PMC8661613

[10] Grywna K, Kupfer B, Panning M, Drexler JF, Emmerich P, Drosten C, et al. Detection of all species of the genus Alphavirus by reverse transcription-PCR with diagnostic sensitivity. J Clin Microbiol. 2010;48:3386–7. 10.1128/JCM.00317-10 20504990 PMC2937745

[11] Forrester NL, Wertheim JO, Dugan VG, Auguste AJ, Lin D, Adams AP, et al. Evolution and spread of Venezuelan equine encephalitis complex alphavirus in the Americas. PLoS Negl Trop Dis. 2017;11:e0005693. 10.1371/journal.pntd.0005693 28771475 PMC5557581

[12] Fischer C, Jo WK, Haage V, Moreira-Soto A, de Oliveira Filho EF, Drexler JF. Challenges towards serologic diagnostics of emerging arboviruses. Clin Microbiol Infect. 2021;27:1221–9. 10.1016/j.cmi.2021.05.047 34111589

[13] Postigo-Hidalgo I, Jo WK, Pedroso C, Brites C, Drexler JF. Introduction of chikungunya virus in coastal northeast Brazil. Lancet Microbe. 2023;4:e764. 10.1016/S2666-5247(23)00176-3 37385284

[14] Carrera JP, Forrester N, Wang E, Vittor AY, Haddow AD, López-Vergès S, et al. Eastern equine encephalitis in Latin America. N Engl J Med. 2013;369:732–44. 10.1056/NEJMoa1212628 23964935 PMC3839813

[15] Moreira-Soto A, Bruno A, de Mora D, Paez M, Garces J, Wulf B, et al. Virological evidence of the impact of non-pharmaceutical interventions against COVID-19 in Ecuador, a resource-limited setting. Emerg Microbes Infect. 2023;12:2259001. 10.1080/22221751.2023.2259001 37698611 PMC10563623

