# Supplementary material for: Venezuelan Equine Encephalitis, Peruvian Amazon, 2020
Source: Emerg Infect Dis. 2025 May;31(5):995–9. doi: 10.3201/eid3105.241694 (PMC12044237; doi:10.3201/eid3105.241694)
Supplement: Appendix — Additional information for Venezuelan equine encephalitis, Peruvian Amazon, 2020. [file 24-1694-Techapp-s1.pdf]

Article DOI: http://doi.org/10.3201/eid3105.241694

EID cannot ensure accessibility for supplementary materials supplied by authors. Readers who have difficulty accessing supplementary content should contact the authors for assistance.

Venezuelan Equine Encephalitis, Peruvian Amazon, 2020

Appendix

Additional Methods

Molecular analyses

Nucleic acids were extracted using the MagNA Pure 96 DNA and Viral NA Small volume kit (https://lifescience.roche.com). Viral loads were quantified using a previously described RT-qPCR assay (*1*). The library was prepared using the KAPA RNA HyperPrep kit (https://sequencing.roche.com), and sequencing was performed on a MiniSeq high-output reagent kit (150 cycles paired-end) (https://www.illumina.com/). The reads were then mapped to the Venezuelan Equine Encephalitis Virus (VEEV) subtype ID reference sequence (GenBank accession number NC_001449) using Geneious (2023.2.1). The genome coverage for the Peru_2020 sequence (11,444 nt; 185,091 reads) and the Peru_2021 sequence (11,432 nt; 14,150,916 reads) was 100%. The raw reads were deposited in the Sequence Read Archive (SRR28743650 and SRR31101754).

Phylogenetic analyses

Nucleotide alignments were done using MAFFT with a G-INS-i algorithm using Geneious (2023.2.1). Bayesian phylogenies were constructed using MrBayes 3.2.6 (https://github.com/NBISweden/MrBayes/releases/tag/v3.2.6) with a general time reversible substitution model with gamma distribution. VEEV reference sequences were retrieved for phylogenetic analysis (https://ictv.global/report/chapter/togaviridae/taxonomy/togaviridae).

Evolutionary analyses

The nucleotide sequence of the complete ORFs was aligned using the G-INS-I algorithm using MAFFT with Geneious software (2023.2.1). The time to the most recent common ancestor (tMRCA) of VEEV was calculated using Bayesian Evolutionary Analysis Sampling Trees (95%

highest posterior density) (BEAST; 1.7.1). The running parameters used for the analysis were similar to those previously described (*2*). These included the substitution model GTR, an uncorrelated relaxed clock with a lognormal distribution and a coalescent Bayesian skyline population growth prior (*2*). The test was run for 10 million generations with 10% of burn-in, sampling every 1000 steps (https://beast.community/tracer). Tip dates were obtained from the sampling year following a previous phylogenetic analysis of VEEV subtype ID and IAB, no further calibration of node ages was performed to construct the tree, with computed node ages slightly older than, but largely comparable to those in the prior study (around 10-20 years difference at most internal nodes) (*2*). Selection pressure analyses for the coding region were done using the single-likelihood ancestor counting implemented in the software package HyPhy (*3*).

Virus isolation

For virus isolation, $1.8x10^5$ Vero E6 cells were seeded per well in 12-well and incubated overnight with Dulbecco's modified Eagle medium (DMEM), supplemented with 10% fetal calf serum (FCS), 1% penicillin-streptomycin (pen/strep) (100 U/mL), and 1% non-essential amino acids (NEAA). The serum sample was diluted 1:25 with 480 µL of DMEM supplemented with 1% FCS. The cells were then inoculated with 250 µL of the sample dilution for 1 hour. The inoculum was replaced with 800 µl of fresh medium. The cells were then incubated at 37°C and 5% CO2 with daily monitoring for cytopathic effects and 50 µl of the supernatant was tested by RT-qPCR assay to confirm viral growth (*1*).

Neutralization assay

The first screening was done using high throughput plaque reduction neutralization test (PRNT), $1.1x10^4$ Vero E6 cells were seeded in 96-well plates and incubated overnight with DMEM supplemented with 10%, FCS, 1%, pen/strep (100 U/mL), and 1% NEAA at 37°C and 5% CO_2. Serum samples and VEEV ID (Peru_2020) were diluted in DMEM supplemented with 1% FCS. For neutralization, 12.5 µl diluted serum were mixed with ~50 plaque forming units (PFU) in a total volume of 25 µl and incubated for one hour at 37°C and 5% CO_2. For inoculation, cell culture medium was removed, and 20 µl serum virus mixture was added per well and incubated for one hour at 37°C and 5% CO_2. Afterwards, 100 µl carboxymethyl cellulose-DMEM (containing 2% FCS) overlay was added per well and cells were incubated for 3 days before fixation and staining with formaldehyde and crystal violet.

Samples that exhibit a neutralization were retested by $PRNT_{50}$ in a 12-well plate, in 2-fold dilution between 1:40–1:320 by PRNT. $1.8x10^5$ Vero E6 cells were seeded in 12-well plates and incubated overnight with DMEM supplemented with 10% FCS, 1% pen/strep (100 U/mL), and 1% NEAA at 37°C and 5% CO_2. Serum samples and VEEV ID (Peru_2020) were diluted in DMEM supplemented with 1% FCS. For neutralization, 34 µl diluted serum were mixed with ~50 plaque forming units (PFU) in a total volume of 68 µl and incubated for one hour at 37°C and 5% CO_2. For inoculation, cell culture medium was removed, and 50 µl serum-virus mixture were added per well and incubated for one hour at 37°C and 5% CO_2. Afterwards, 1.5 mL of carboxymethyl cellulose-DMEM (containing 2% FCS) overlay were added per well and cells were incubated for 3 days before fixation and staining with formaldehyde and crystal violet.

Of the 53 sera that showed neutralization in the screening test, 35 were negative in the titration test. This may be due to the difficulty in performing an adequate count of PFU in the 96-well plates. To maintain the robustness of the analysis, only sera that showed neutralization in the titration were considered for IFA. The limited amount of serum available did not allow us to perform further analysis on the negative sera.

Immunofluorescence assay (IFA)

An indirect immunofluorescence assay (IFA) was conducted to detect IgM/IgG antibodies to VEEV. The assay was performed using a commercially available IFA kit (https://www.euroimmun.com/) based on cells infected with VEEV, Chikungunya, O'nyong-nyong, Ross River, Western equine encephalitis, Sindbis, Barmah Forest, Eastern Equine Encephalitis viruses and on virus-like particles for Mayaro and Chikungunya virus. Briefly, serum samples were diluted 1:10 for IgM, in immunoadsorbent Eurosorb to deplete class IgM rheumatoid factors and deplete IgG antibodies and 1:100 for IgG in dilution buffer. Samples were then added to the slide and incubated for 30 minutes at room temperature, followed by a washing step with PBS-Tween. Subsequently, a secondary IgM or IgG antibody coupled to fluorescein was added and incubated for 30 minutes at room temperature. Subsequently, a second wash was conducted. The results of IFA were visualized and photographed using a Leica DMi8 microscope.

References

1. Carrera JP, Araúz D, Rojas A, Cardozo F, Stittleburg V, Morales Claro I, et al. Real-time RT-PCR for Venezuelan equine encephalitis complex, Madariaga, and Eastern equine encephalitis viruses: application in human and mosquito public health surveillance in Panama. J Clin Microbiol. 2023;61:e0015223. PubMed https://doi.org/10.1128/jcm.00152-23

2. Forrester NL, Wertheim JO, Dugan VG, Auguste AJ, Lin D, Adams AP, et al. Evolution and spread of Venezuelan equine encephalitis complex alphavirus in the Americas. PLoS Negl Trop Dis. 2017;11:e0005693. PubMed https://doi.org/10.1371/journal.pntd.0005693

3. Pond SL, Frost SD, Muse SV. HyPhy: hypothesis testing using phylogenies. Bioinformatics. 2005;21:676–9. PubMed https://doi.org/10.1093/bioinformatics/bti079

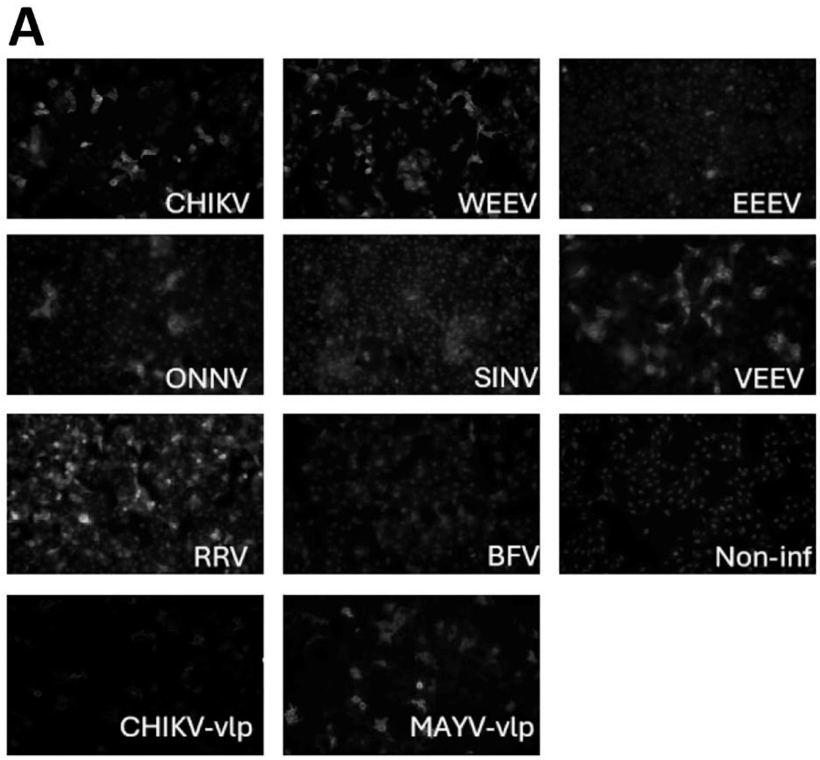

B

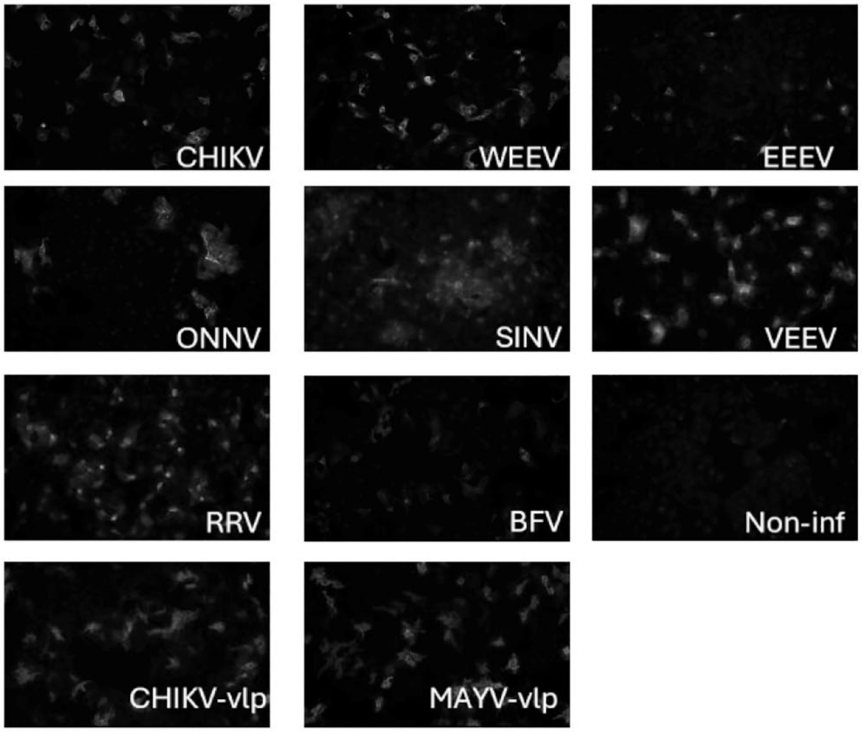
CHIKV
WEEV
EEEV
ONNV
SINV
VEEV
RRV
BFV
Non-inf
CHIKV-vlp
MAYV-vlp

C

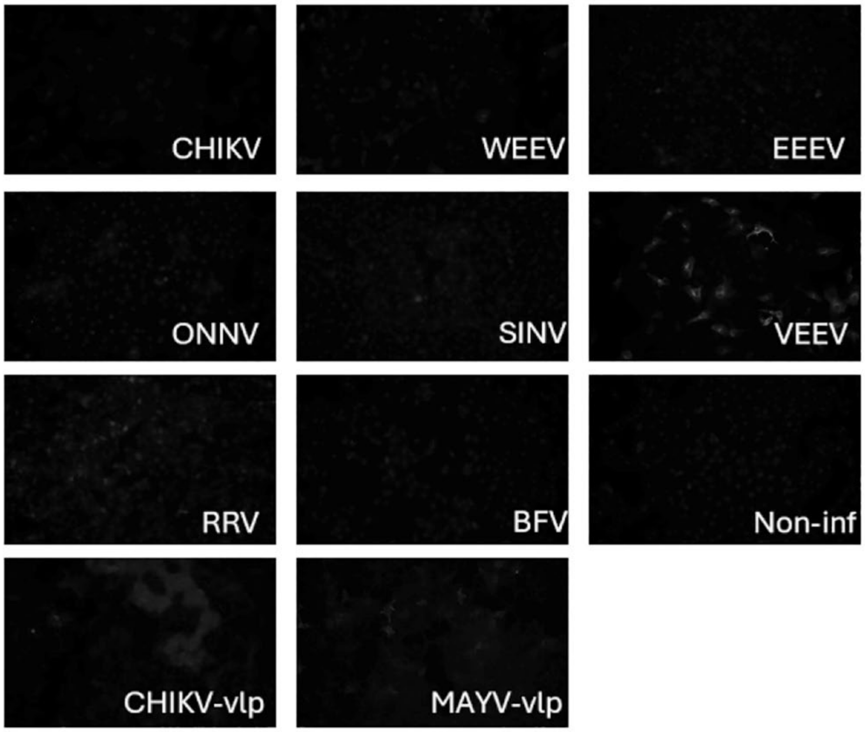
CHIKV
WEEV
EEEV
ONNV
SINV
VEEV
RRV
BFV
Non-inf
CHIKV-vlp
MAYV-vlp

D

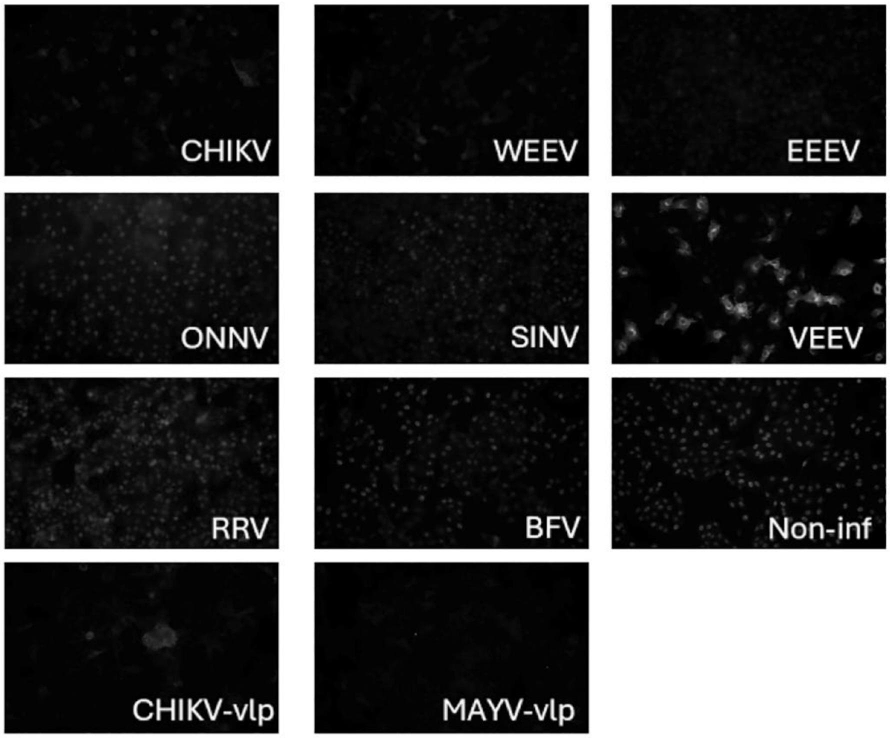
CHIKV
WEEV
EEEV
ONNV
SINV
VEEV
RRV
BFV
Non-inf
CHIKV-vlp
MAYV-vlp

E

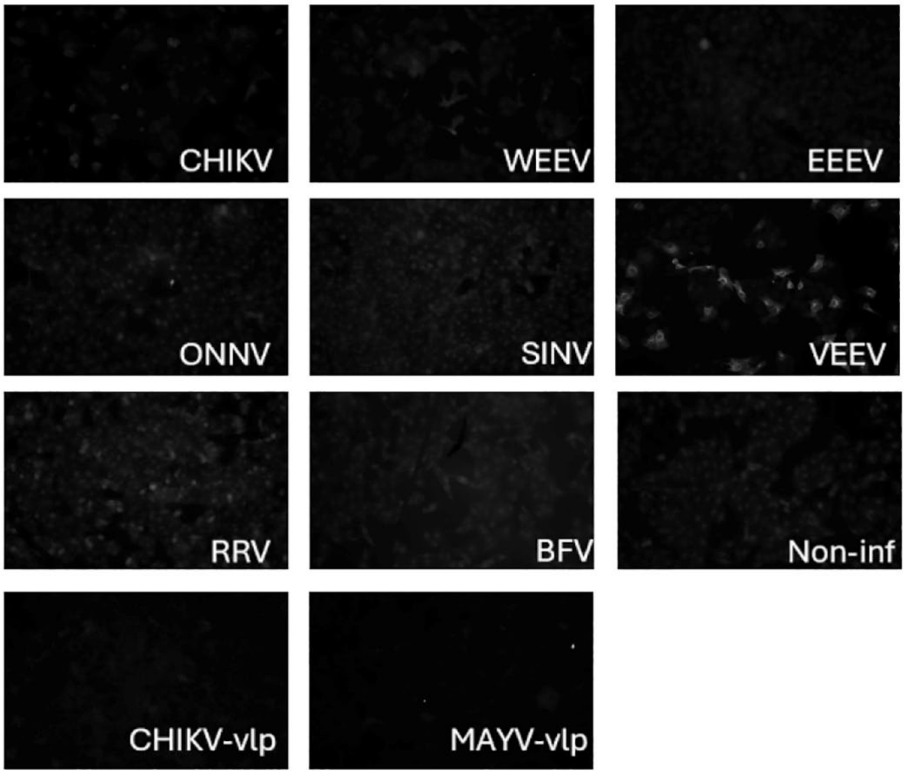
CHIKV
WEEV
EEEV
ONNV
SINV
VEEV
RRV
BFV
Non-inf
CHIKV-vlp
MAYV-vlp

F

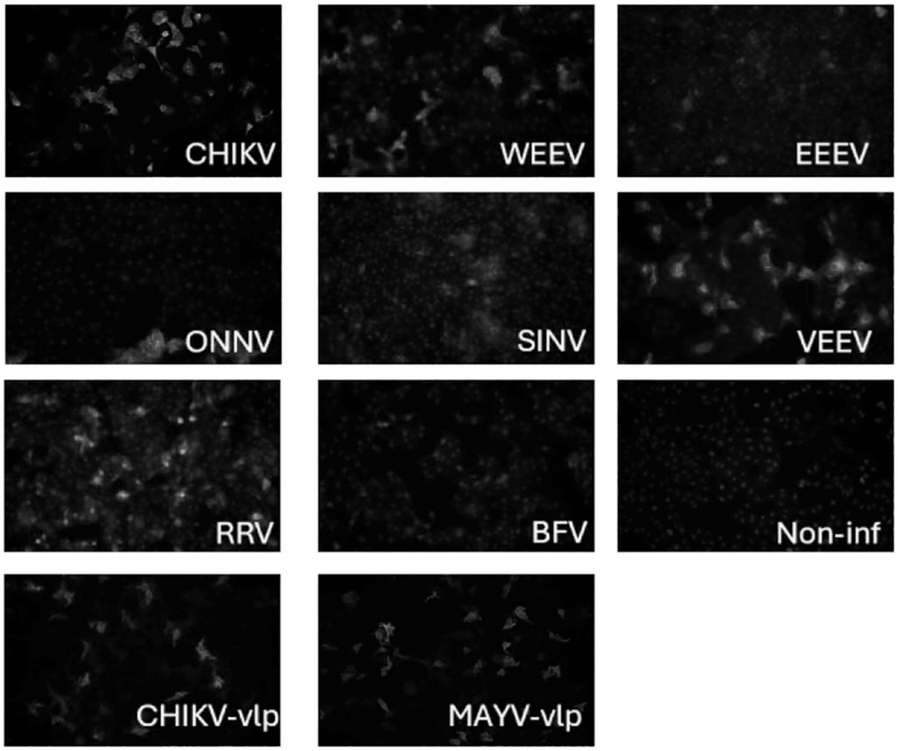
CHIKV
WEEV
EEEV
ONNV
SINV
VEEV
RRV
BFV
Non-inf
CHIKV-vlp
MAYV-vlp

G

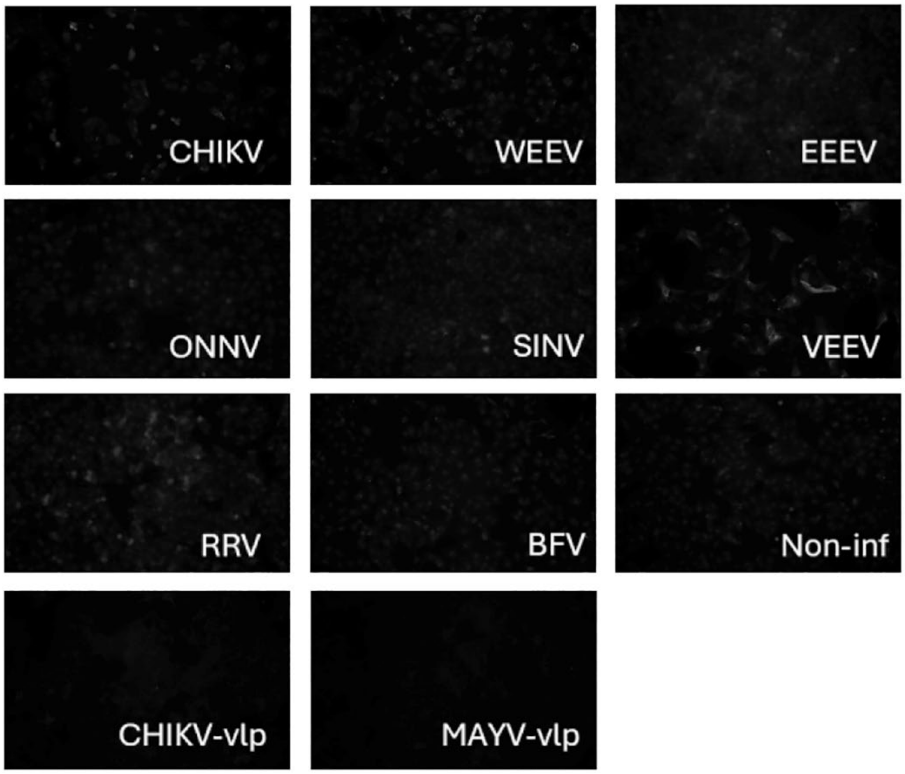
CHIKV
WEEV
EEEV
ONNV
SINV
VEEV
RRV
BFV
Non-inf
CHIKV-vlp
MAYV-vlp

H

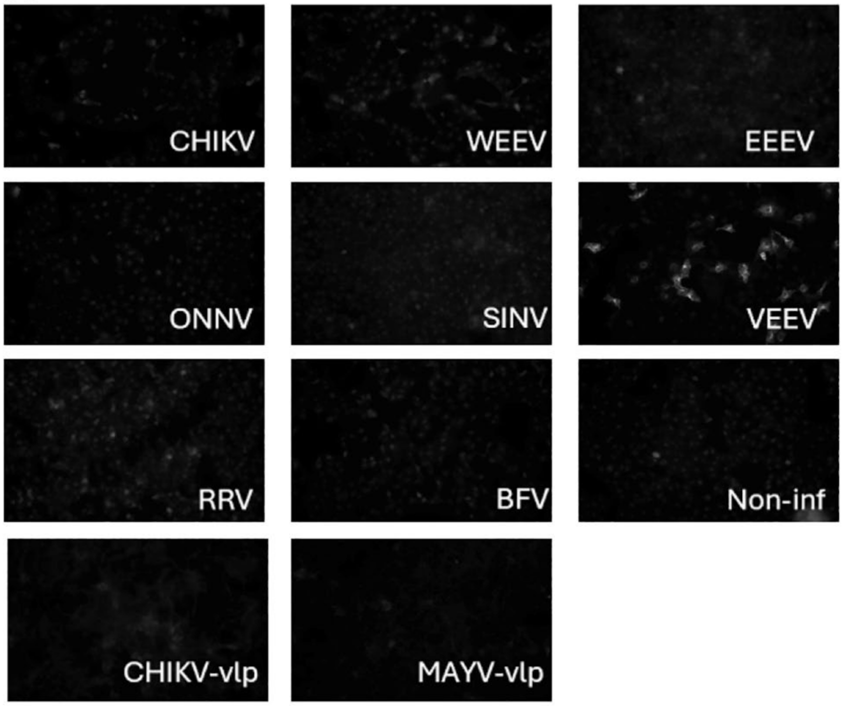
CHIKV
WEEV
EEEV
ONNV
SINV
VEEV
RRV
BFV
Non-inf
CHIKV-vlp
MAYV-vlp

I

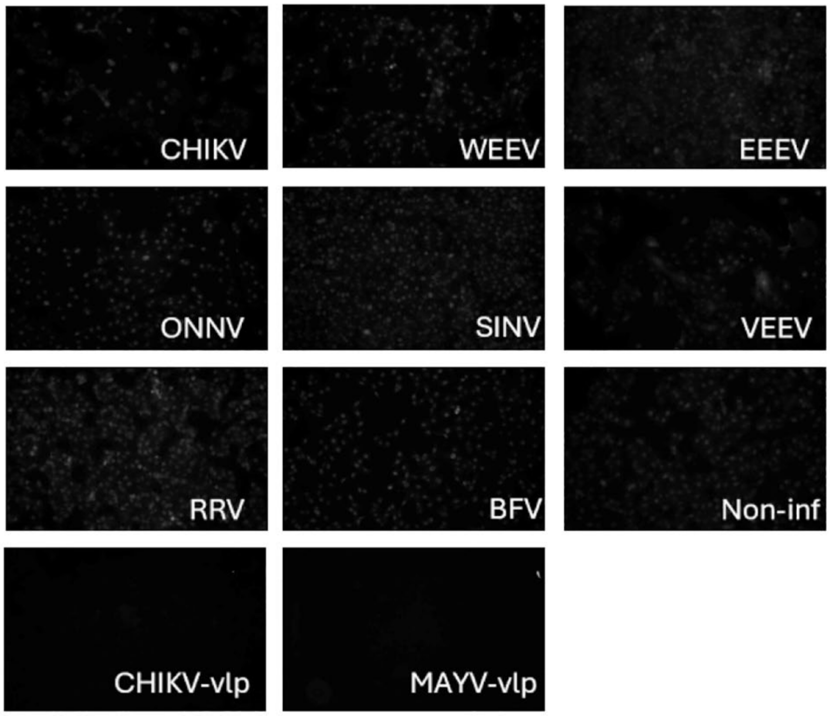
CHIKV
WEEV
EEEV
ONNV
SINV
VEEV
RRV
BFV
Non-inf
CHIKV-vlp
MAYV-vlp

J

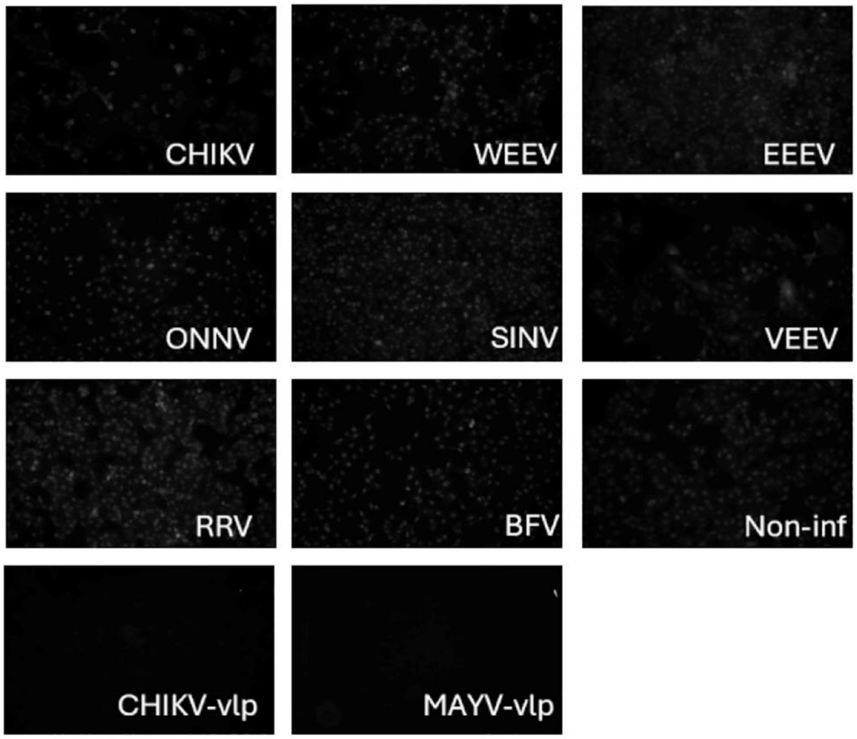
CHIKV
WEEV
EEEV
ONNV
SINV
VEEV
RRV
BFV
Non-inf
CHIKV-vlp
MAYV-vlp

K

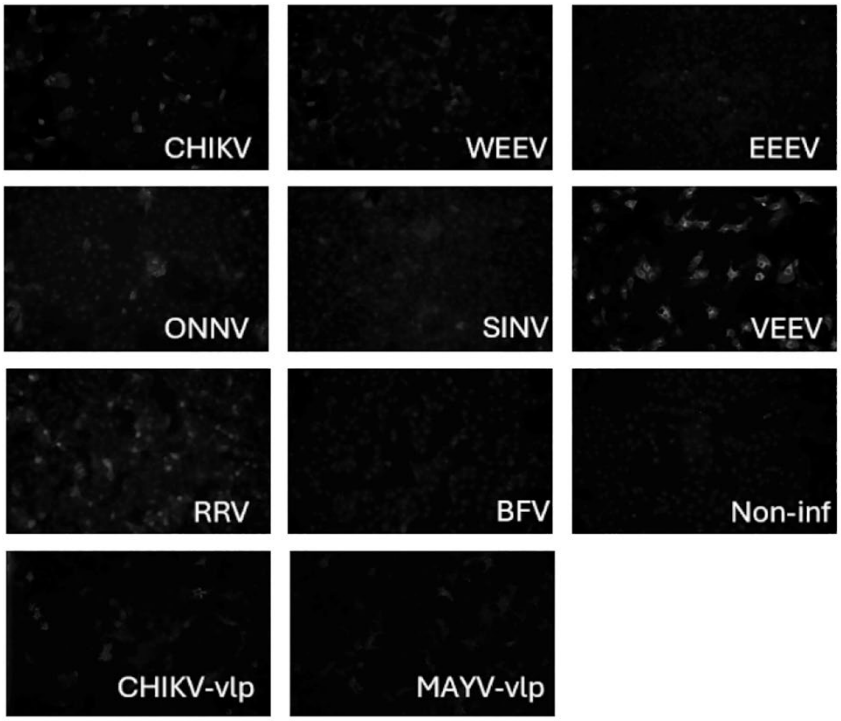
CHIKV
WEEV
EEEV
ONNV
SINV
VEEV
RRV
BFV
Non-inf
CHIKV-vlp
MAYV-vlp

L

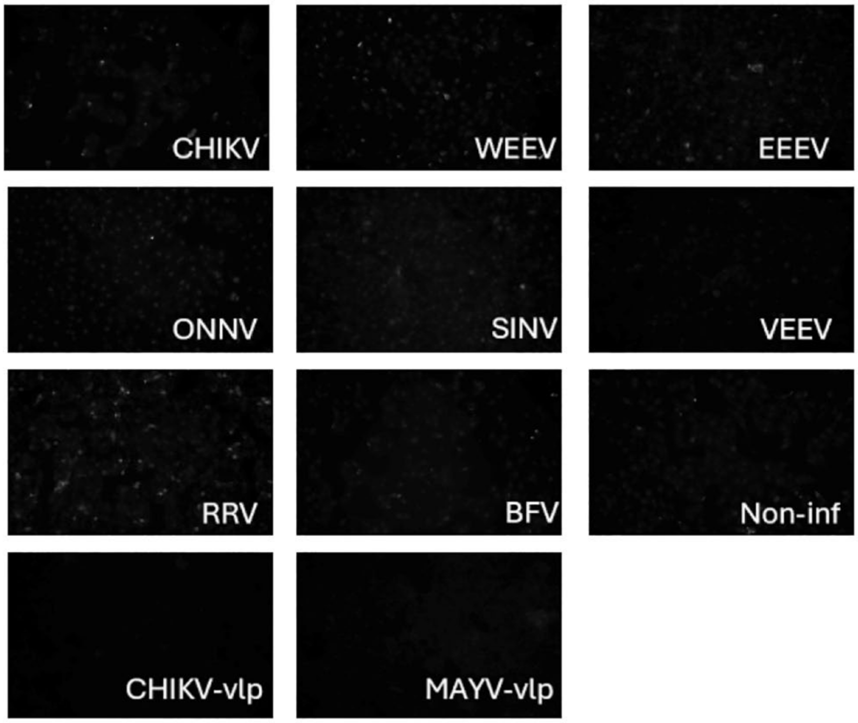
CHIKV
WEEV
EEEV
ONNV
SINV
VEEV
RRV
BFV
Non-inf
CHIKV-vlp
MAYV-vlp

M

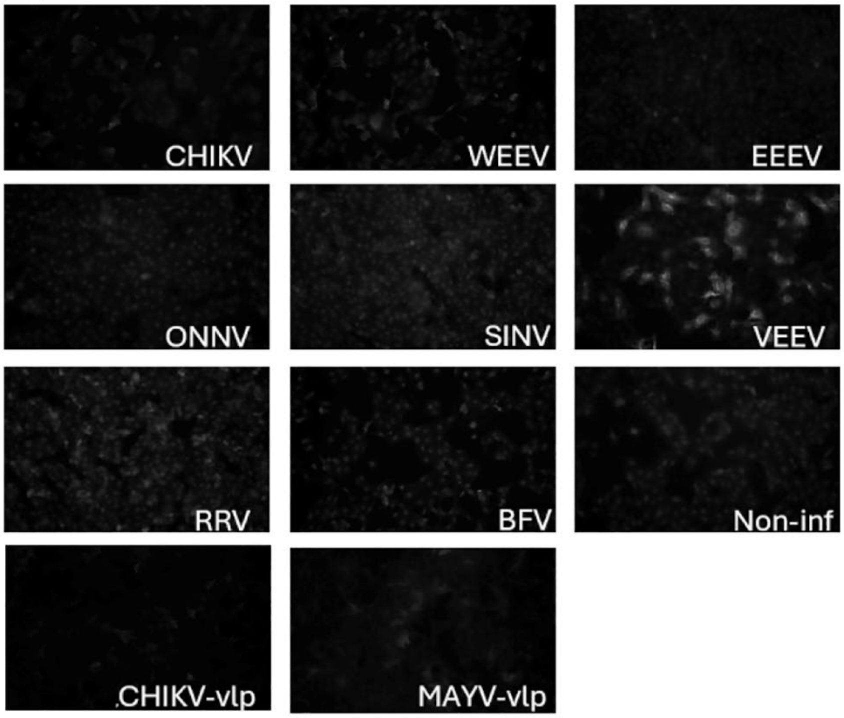
CHIKV
WEEV
EEEV
ONNV
SINV
VEEV
RRV
BFV
Non-inf
CHIKV-vlp
MAYV-vlp

N

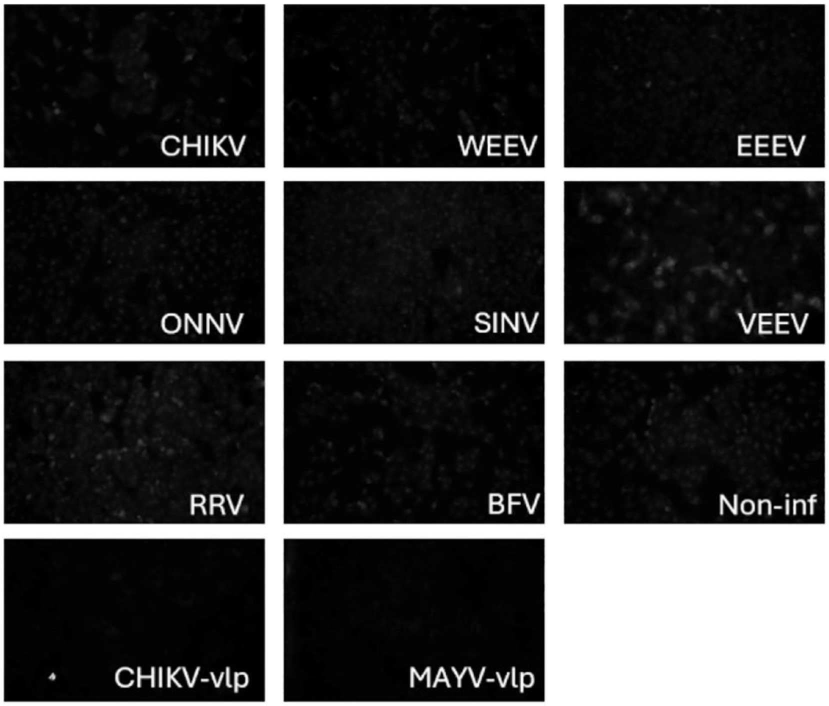
CHIKV
WEEV
EEEV
ONNV
SINV
VEEV
RRV
BFV
Non-inf
CHIKV-vlp
MAYV-vlp

O

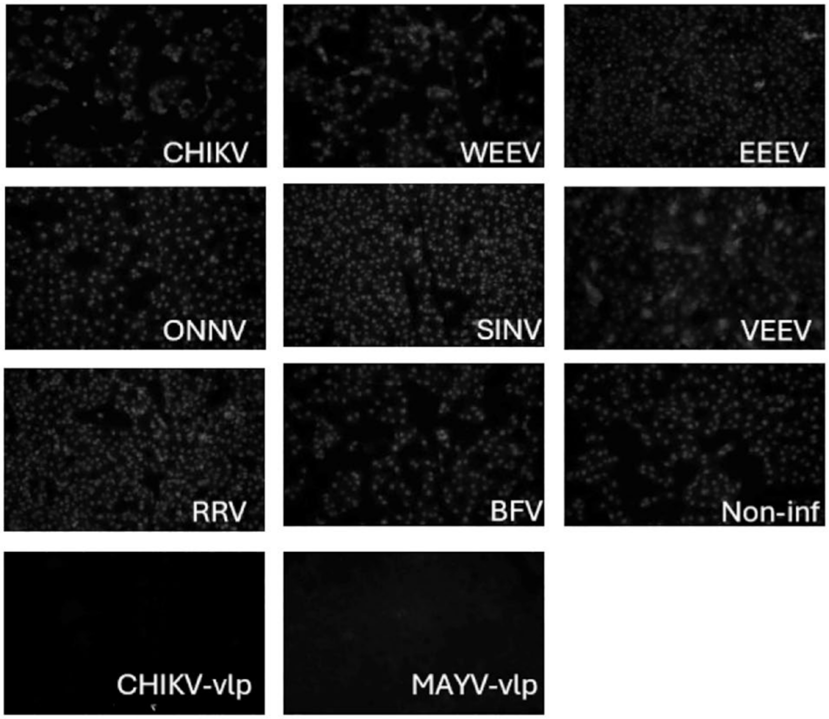
CHIKV
WEEV
EEEV
ONNV
SINV
VEEV
RRV
BFV
Non-inf
CHIKV-vlp
MAYV-vlp

P

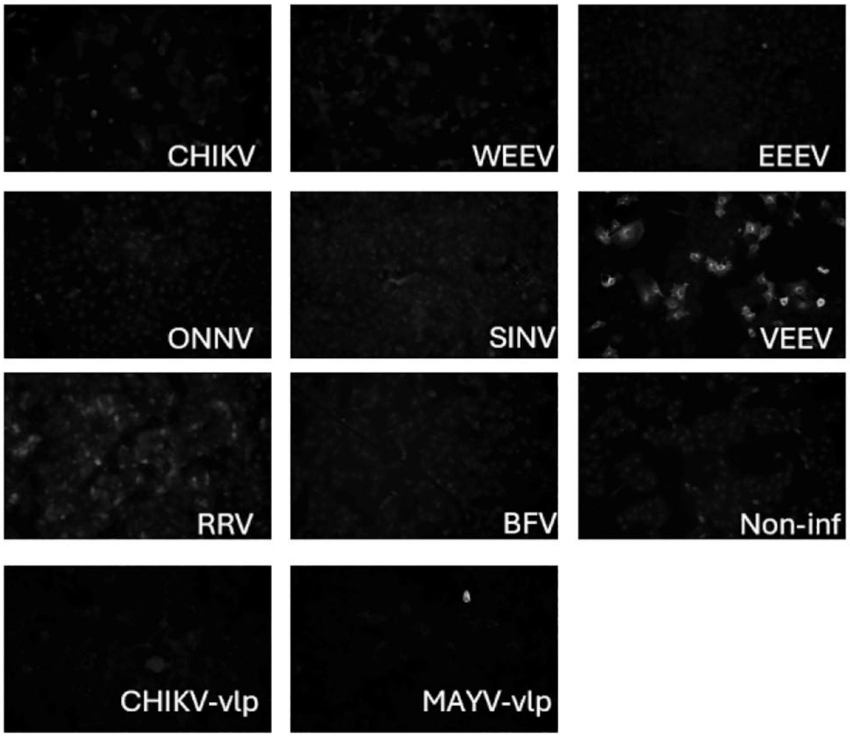
CHIKV
WEEV
EEEV
ONNV
SINV
VEEV
RRV
BFV
Non-inf
CHIKV-vlp
MAYV-vlp

Q

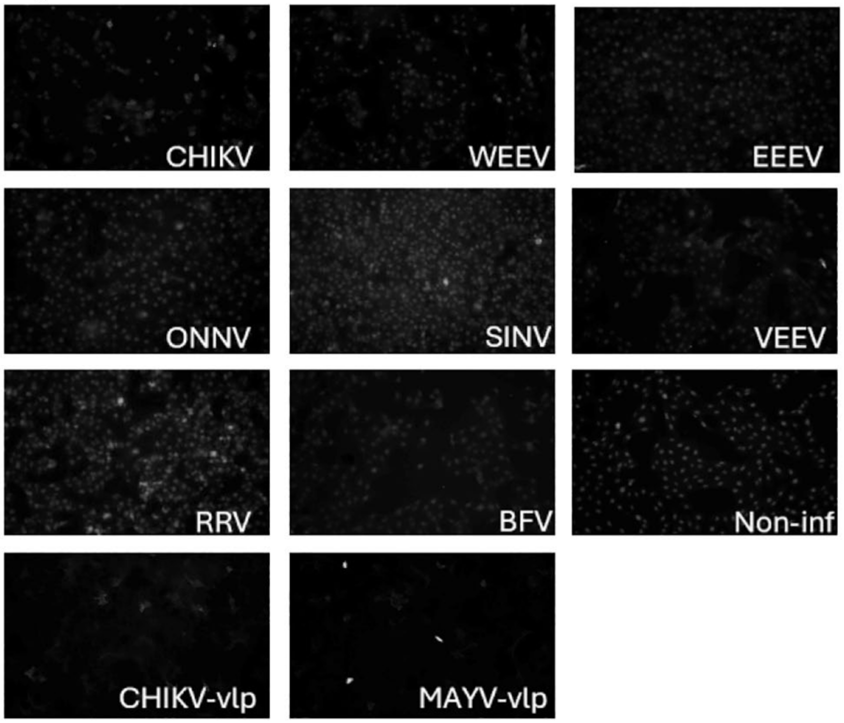
CHIKV
WEEV
EEEV
ONNV
SINV
VEEV
RRV
BFV
Non-inf
CHIKV-vlp
MAYV-vlp

R

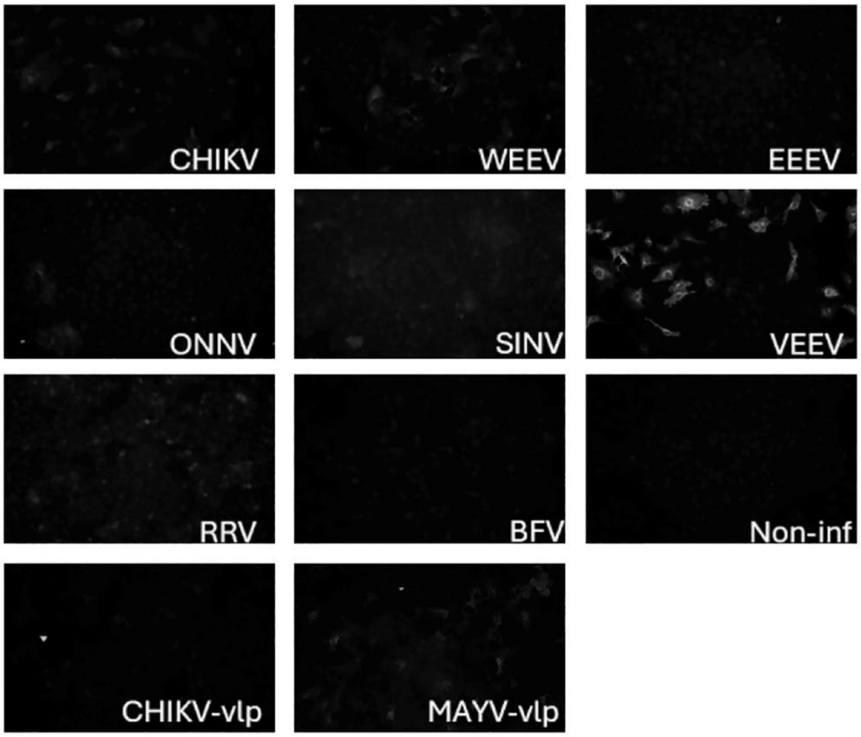

Appendix Figure 1. Immunofluorescence assay for IgG antibodies to alphaviruses: Venezuelan equine encephalitis virus $PRNT_{50}$–positive serum samples. Data are shown by sample ID: (A) 3216, (B) 3270, (C) 3393, (D) 3399, (E) 3624, (F) 3634, (G) 3876, (H) 3927, (I) 3940, (J) 4140, (K) 4294, (L) 4321, (M) 4420, (N) 4590, (O) 4669, (P) 4747, (Q) 4768, and (R) 4797. CHIKV, Chikungunya virus; ONNV, O'nyong-nyong virus; RRV, Ross River virus; BFV, Barmah Forest virus; SINV, Sindbis virus; WEEV, Western equine encephalitis virus; EEEV, Eastern equine encephalitis virus; VEEV, Venezuelan equine encephalitis virus; MAYV, Mayaro virus; VLP, virus-like particles. All serum samples were tested 1:100.

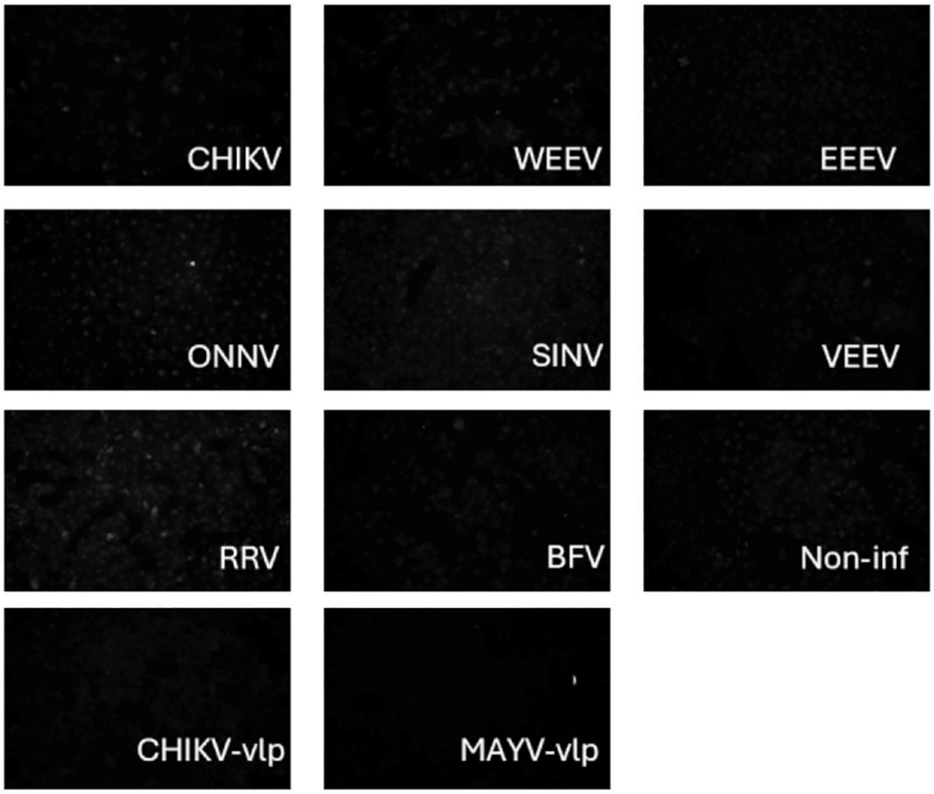

Appendix Figure 2. Immunofluorescence assay for IgM antibodies to alphaviruses: example of a negative serum sample. CHIKV, Chikungunya virus; ONNV, O'nyong-nyong virus; RRV, Ross River virus; BFV, Barmah Forest virus; SINV, Sindbis virus; WEEV, Western equine encephalitis virus; EEEV, Eastern equine encephalitis virus; VEEV, Venezuelan equine encephalitis virus; MAYV, Mayaro virus; VLP, virus-like particles. Serum samples were tested 1:10.
